# $\dot{V}$ O_2_ linear-onset kinetics spanning steady- and non-steady-state exercise

**DOI:** 10.3389/fphys.2025.1547662

**Published:** 2025-08-15

**Authors:** Robert Robergs, Bridgette O’Malley, Anais Dewilde, Shaun D’Auria, Ales Krouzecky

**Affiliations:** ^1^ School of Exercise and Nutrition Sciences, Faculty of Health, Queensland University of Technology, Kelvin Grove, QL, Australia; ^2^ Faculty of Health Studies, Jan Evangelista Purkyne University, Usti nadLabem, Czechia; ^3^ Department Of Sports Medicine and Active Health Sciences, Faculty of Medicine, Charles University, Pilsen, Czechia; ^4^ Queensland Academy Of Sport, Nathan, QL, Australia

**Keywords:** cycling, endurance exercise, training, plateau, critical power, oxygen consumption

## Abstract

**Introduction:**

The traditional method for quantifying the kinetics of the increase in the body’s consumption of oxygen ( $\dot{V}$ O_2_) during exercise transitions to steady state involves application of a mono-exponential function. Anomalies exist to question the validity of this method, as they show the initial (∼1 min) of this $\dot{V}$ O_2_ response is linear.

**Methods:**

Fourteen highly endurance trained subjects (12 males, 2 females) completed a ramp incremental cycling protocol, as well as 8 different constant load trials at 43 to 148 % of their critical power (CP).

**Results:**

For the initial five exercise bouts, the linear fit of the initial segment was significantly more accurate (lower standard error of estimates; SE) compared to the mono-exponential fit (p < 0.001). There were two different systematic profiles of the linear onset (LO) $\dot{V}$ O_2_ slope from different bouts of increasing exercise intensities; 1) a sustained increase (increased kinetics) (n = 7), and 2) a plateau or decrease (impaired kinetics) (n = 7). Both sub-groups were similar in all measures of cardio-respiratory and muscular endurance.

**Discussion:**

The LO $\dot{V}$ O_2_ kinetics method is superior to the traditional approach as it was a more valid representation of the initial $\dot{V}$ O_2_ response, can be applied to both steady and non-steady state exercise intensities, requires less than 2 min of exercise, but across multiple bouts, and identifies more complex physiology than the mono-exponential method. Added research is needed to discern the most valid methods to measure LO $\dot{V}$ O_2_ kinetics, and to learn more about its physiological determinants compared to the traditional mono-exponential method.

## Introduction

As this research presents evidence for application of a new method for studying the whole body’s consumption of oxygen ( $\dot{V}$ O_2_) kinetic response to the onset of a higher intensity of exercise, there is a need to concisely document the historical development of the study of $\dot{V}$ O_2_ kinetics. Research on the rate of increase in $\dot{V}$ O_2_ during exercise transitions to steady state has a long history. The first cited research on this topic was in 1913, where crude estimations of $\dot{V}$ O_2_ from end-tidal air samples were graphically presented along with non-linear functions of this response, in addition to ventilation and heart rate (Krogh and Lindhard, 1913). These authors did not refer to a mono-exponential model or explain the curve fitting to their data, but regardless of the poor control over exercise intensity for each bout, the data presentation for specific exercise trials reaching a steady state revealed such a response. Few subjects were used in the research (n = 6), although the methodology was more akin to a single-subject design of different exercise methods. In addition, few data points were used to profile the $\dot{V}$ O_2_ responses (n = 5–7).

Hill et al. (1923) and Hill et al. (1924) applied mono-exponential curve fitting to their $\dot{V}$ O_2_ data acquired during the transition of running from rest to different low-to-moderate exercise intensities, which each elicited a steady-state response, although the authors did not comment on this data processing or on the small size of the participant sample (n = 5) and datasets used to profile individual $\dot{V}$ O_2_ responses to constant-load exercise bouts (n = 3 to 7 data points). Most of the researchers who followed Hill and Lupton’s results for the next 20 years focused on the kinetics of recovery $\dot{V}$ O_2_ and, as such, further tested the concept of O_2_ debt (Henry, 1951; Margaria et al., 1965; Henry and DeMoor, 1950; Wells et al., 1957; Di Prampero et al., 1970; Whipp, 1971).

The earliest study we could find that provided a mathematical function for the non-linear increase in $\dot{V}$ O_2_ for a constant intensity exercise bout was Henry (1951); the function is presented in Equation 1. However, the focus of this prior research, once again, was not on the exercise induced $\dot{V}$ O_2_ response but the post-exercise recovery, and subject numbers (n = 10) and data points (n = 7) for each individual kinetic analysis and non-linear profiling were small (see their Figure 3, p. 435).

$${\dot{V}O}_{2}=a_{0}\left(1-e^{-kt}\right).$$ (1)

Margaria et al. (1965) presented a similar equation for the non-linear $\dot{V}$ O_2_ response to exercise transitions to steady state (Equation 2) based on small subject numbers (n = 4) and data points for given exercise conditions (n = 11).

$${\dot{V}O}_{2}=10a\left(1-10^{-kt}\right),$$ (2)

where $\dot{V}$ *O*_2_ = $\dot{V}$ *O*_2_ *increment*; 10*a* = e*xercise intensity expressed as* $\dot{V}$ *O*_2_; k = *velocity constant*; *t* = *time* (*s*)

In the 1960s, despite major limitations in research design, sample sizes, small datasets per exercise condition resulting from instrumentation and method constraints, and no rigorous elucidation of competing model scenarios, the mono-exponential model of this $\dot{V}$ O_2_ response (Henry, 1951; Margaria et al., 1965; Henry and DeMoor, 1950; Wells et al., 1957; Di Prampero et al., 1970; Whipp, 1971; Dale and Glaister, 2018) was accepted. This was best observed in the acceptance of the mono-exponential model by Whipp (1971) based on the previously cited research concerning constrained methodologies and participant sample sizes (Henry, 1951; Margaria et al., 1965; Henry and DeMoor, 1950; Wells et al., 1957). Nevertheless, Whipp (1971) further improved on the mathematical and graphical data processing for $\dot{V}$ O_2_ data acquired from gas exchange during exercise increments to steady state (Equation 3).

$${\dot{V}O}_{2\left(t\right)}={\dot{V}O}_{2\left(ss\right)}\left(1-e^{-kt}\right),$$ (3)

where $\dot{V}$ *O*_2(t)_ = $\dot{V}$ *O*_2_ *increment*; $\dot{V}$ O_2(ss)_ = *steady state* $\dot{V}$ *O*_2_; *k* = *rate constant*; *t* = *time* (*min*)

Consequently, during this time period there were numerous limitations to the establishment of the mono-exponential model of the $\dot{V}$ O_2_ kinetics to exercise transitions. For example, the main exercise condition of interest was steady state, non-linear modelling was based on only one method (mono-exponential), and for the prior 50 years, such a model was developed from rudimentary methods and insufficient data sets (∼5 data points over 3 min).

**FIGURE 1:**
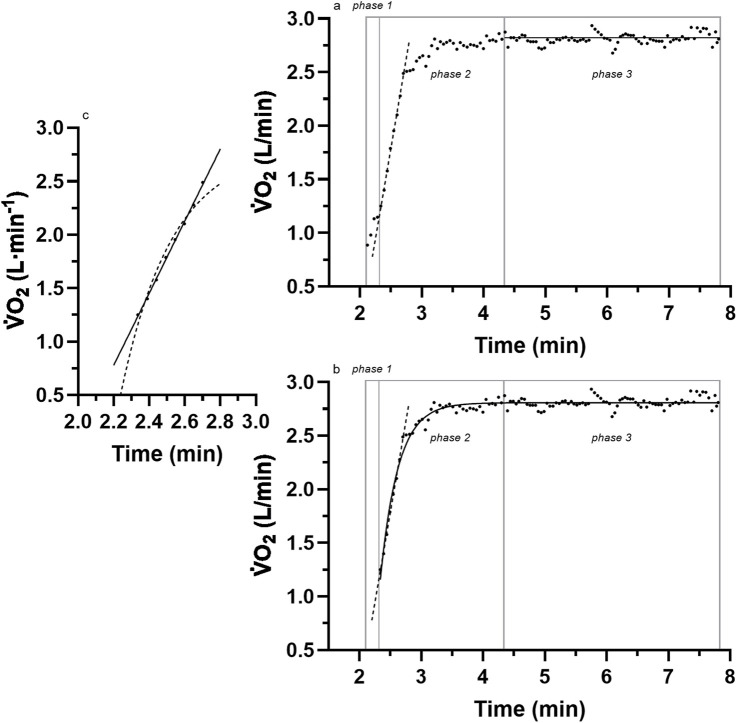
A sample of breath-by-breath $\dot{V}$ O_2_ data of the three different phases of the kinetic response for **(a)** the linear onset fit; **(b)** a comparison to the traditional mono-exponential fit of the entire data; **(c)** a closer look at the linear vs. exponential fit of the linear-onset segment. Adapted from McNulty and Robergs (2017). See the text for details.

Considerable research exists on $\dot{V}$ O_2_ kinetics for exercise transitions to steady state since this time, but the principle of applying a mono-exponential fit to data for exercise bouts approximating 5–10 min from a lower to a higher steady-state intensity has remained core to the method (Dale and Glaister, 2018; Whipp and Wasserman, 1972; Koppo et al., 2004). Consequently, the mono-exponential assumption of the $\dot{V}$ O_2_ kinetic response to exercise transitions to steady state has remained until now, albeit slightly improved to denote minor corrections (e.g., removal of baseline $\dot{V}$ O_2_) and added components (e.g., the initial time delay, TD) of the $\dot{V}$ O_2_ response (Whipp, 1971) (Equation 4) (Figure 1b).

$${∆\dot{V}O}_{2\left(t\right)}={∆\dot{V}O}_{2}\left(1-e^{-\left[\left(t-TD\right)/\tau \right]}\right),$$ (4)

where ∆ $\dot{V}$ O_2_(t) = exercise $\dot{V}$ O_2_ − baseline $\dot{V}$ O_2_; TD = initial time delay; τ = time constant; t = time (min)

The persistent acceptance of the mono-exponential model is unfortunate because considerable research has produced inconsistent data (anomalies) with such a model. Whipp and Wasserman (1972) presented representative raw data from one of their subjects and revealed linear responses for the initial 1 min of the exercise transition $\dot{V}$ O_2_ data (see their Figure 1, p. 352). These linear responses would have been even more apparent had the authors deleted the initial data (now known as phase-1, which reveals an initial exaggerated kinetic response) presently labeled as a cardio-pulmonary adjustment (Koppo et al., 2004). More clear evidence of this initial linear kinetic response of $\dot{V}$ O_2_ to exercise transitions was presented by Diamond and colleagues (see their Figure 1, p. 705) Diamond et al. (1977). Once again, these data were anomalies to the mono-exponential model, were ignored (perhaps not seen), and therefore adhered to Kuhn (1962)’s dire observations of “normal” science constrained by excessive reliance on and the inability to challenge conventional methodologies. For example, in his own words, “*No part of the aim of normal science is to call forth new sorts of phenomena; indeed those that will not fit the box are often not seen at all.*” (p. 24).

More recent evidence of anomalies to the initial linear segment of the mono-exponential model were reported by Hughson and Morrissey (1982) (see their Figure 2, and 2 p. 923,924) and Poole and Jones (2012) (see their Figure 5, p. 5; Figure 9, p.11), although in both instances, the anomalies remained undetected. Unfortunately, the identification and scientific investigation of the linear onset anomalies did not occur until Robergs (2014) critically commented on the methods used in research of the $\dot{V}$ O_2_ kinetics of exercise transitions, and then McNulty and McNulty and Robergs (2017) documented the superior fit of a linear (vs. mono-exponential) model to the initial onset segment of data after removal of the initial cardio-pulmonary dynamic phase (Figures 1a–c).

**FIGURE 2:**
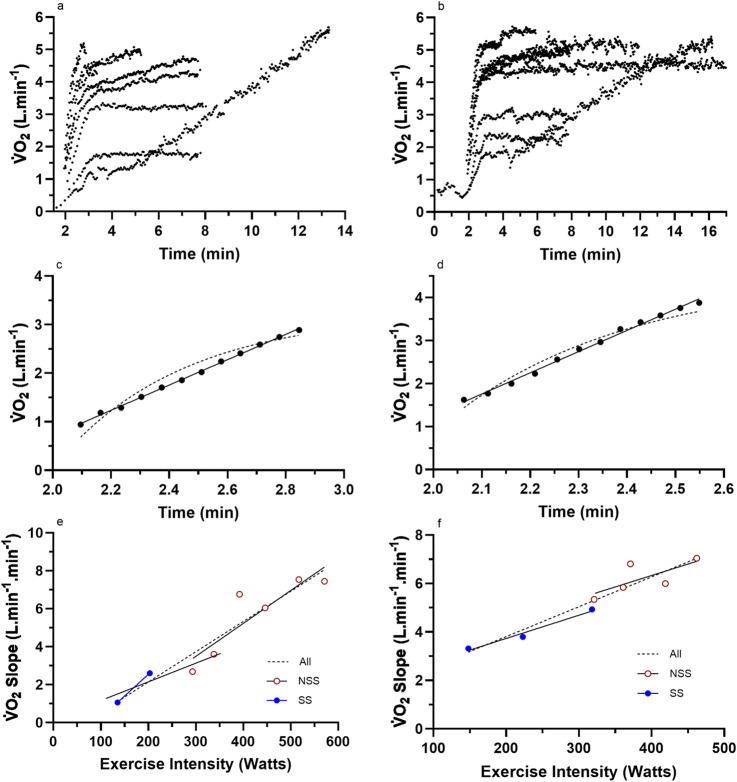
Representative data from two subjects for **(a,b)** the nine (incremental exercise to $\dot{V}$ O_2max_ + eight constant intensity) cycling bouts; **(c,d)** an example bout to show the difference in linear vs. mono-exponential fit for the LO segment; **(e,f)** the total processed LO slope data for each subject across the eight different constant-intensity exercise bouts. For these two subjects, the increasing $\dot{V}$ O_2_ slope with an increase in the exercise intensity adhered to a linear profile. NSS, non-steady-state exercise bouts; SS, steady-state exercise bouts; All, linear or non-linear regression of the combined dataset.

Consequently, the purpose of this research was to further investigate the linear onset $\dot{V}$ O_2_ kinetics phenomenon to assess the best model that fits this segment, to document the presence of this segment during exercise transitions across steady and non-steady state intensities, and if pertinent, explore the added physiology that this new method has been able to discover.

## Methods

All procedures of this research were screened and approved by the University Human Research Ethics Committee (UHREC) (ethics number 4252), which adheres to all pertinent requirements of the Declaration of Helsinki applicable to medical and non-medical research on human participants.

### Participants

Prior data analyses of different physiological topics concerning this research have been published elsewhere (O’Malley et al., 2024). Participants were recruited through social media advertising and were required to be currently completing at least three 45-min cycling endurance sessions per week. Post-recruitment required further evaluation of the subjects to ascertain whether certain additional inclusion and exclusion criteria were met. Such criteria involved the absence of cardiopulmonary, metabolic, and musculoskeletal diseases or conditions, any musculoskeletal injuries, or surgical procedures within 3 months of data collection. This was aided by the completion of the Australian Adult Pre-Exercise Screening System (APSS) questionnaire (Exercise Is Medicine - Australia, 2024). Male and female participants were required to be aged 18–45 and 18–55 years, respectively, as per ACSM maximal exercise testing guidelines (Liguori et al., 2022). Subjects were also required to have no prior history or current use of tobacco smoking.

After this evaluation, 14 (12 male and 2 female) healthy subjects (Table 1) were required to provide their written informed consent to participate in the upcoming exercise trials of the study. According to preliminary sample size estimation (*a priori*), a total of 12 participants were deemed necessary, considering an estimated effect size of 0.5, statistical power of 0.8, and α level of 0.05 (GPower, v3.1.9.4, Universität Kiel, Germany). Nevertheless, the sample size was set at 14 to accommodate potential instances of missing data or participant withdrawal from the study.

**TABLE 1:** Descriptive characteristics of the subjects.

Subject #	Age (yrs)	Sex (M/F)	Height (cm)	Mass (kg)	$\dot{V}$ O_2max_ (ml·kg^-1^·min^-1^)	$\dot{V}$ O_2max_ (Watts)
1	42	M	174.1	87.45	67.41	460
2	29	M	187.0	73.85	76.84	451
3	43	M	175.0	69.60	73.14	400
4	43	M	184.9	85.4	63.38	495
5	46	F	172.4	69.45	54.71	354
6	34	M	190.6	97.30	60.74	484
7	47	F	159.2	51.40	43.38	223
8	35	M	182.1	82.05	60.55	432
9	41	M	192.2	88.85	55.60	461
10	30	M	173.5	71.15	59.56	355
11	34	M	183.2	76.80	57.57	425
12	36	M	177.9	68.10	71.54	472
13	40	M	188.8	83.65	51.70	388
14	43	M	188.8	91.80	52.88	370
Mean ± SD	**38.78** ± **5.75**	-	**180.69** ± **9.17**	**78.35** ± **12.05**	**60.64** ± **9.20**	**412** ± **72**

### Testing protocols

All tests were conducted within the exercise physiology laboratory of the Institute of Health and Biomedical Innovation (IHBI) at the Queensland University of Technology. As previously explained by O’Malley et al. (2024), the testing protocol was conducted over 4 days and consisted of one familiarization session, a continuous incremental cycle ergometer ramp protocol to maximal exertion for the measurement of the maximal rate of oxygen consumption ( $\dot{V}$ O_2max_), four low-to-moderate constant-load (LMCL) tests, and four high-to-severe constant-load (HSCL) tests to quantify the critical power (CP) of the participants.

#### Familiarization session

This protocol introduced subjects to the research laboratory and involved a briefing on the exercise protocols, measurement of the subjects’ age (years), body mass (kg), height (cm), fitness level (low, moderate, and high), and resting heart rate (beats⋅min^-1^), and the completion of a $\dot{V}$ O_2max_ test. Prior to the $\dot{V}$ O_2max_ test, subjects were fitted with an appropriate one-way valve mouthpiece attached to an acrylic head unit to minimize any risk of lost gas exchange data due to an inappropriately sized mouth and headpiece.

#### $\dot{V}$ O_2max_ test

Prior to the commencement of the $\dot{V}$ O_2max_ test, subjects were first seated on the cycle ergometer during which gas exchange data (expired gases) were measured for 2 min. Following this, subjects were instructed to complete 2 min of cycling at a two-fold intensity equivalent to the subjects’ predetermined ramp protocol, followed by a continuous ramp function (Watt increment applied at ∼0.5 Hz) to exercise intolerance. The aforementioned ramp protocol was established from the subjects’ self-reported fitness level to reach volitional exhaustion within 8–12 min. Exercise intolerance was defined as the subject being unable to maintain a cadence at less than 20 rev⋅min^-1^ below their set cadence or through subject self-selected exercise termination.

Heart rate and electrocardiography (ECG) data were collected to monitor any adverse cardiovascular events as a direct result of severe exercise testing protocols and were measured through a 5-lead ECG configuration (Custo-Med, Ottobrunn, Germany). Post-testing protocols required the subject to perform low-intensity (∼50 W) cycling for 2 min on a cycle ergometer as an active cool-down. The subject was then instructed to dismount the ergometer and lie supine for a period of 10 min to support recovery.

#### LMCL and HSCL tests

All constant-intensity exercise bouts were distributed over 3 days with a minimum of 24 h between the days of testing. The LMCL bouts always preceded the HSCL bouts, and the four bouts of each category were administered in a Latin Squares order across all participants (a sequentially different order of bouts across subjects to vary the test order between subjects). On day 2 of testing (24 h following $\dot{V}$ O_2max_ test), participants returned to complete two LMCL tests at either 30, 45, 56, or 75% of their peak power output (W_peak_) (as determined using the $\dot{V}$ O_2max_ test; see the *Measurements* section) and one HSCL test at a predetermined percentage of the participants’ calculated power output at the ventilatory threshold (VT) (see the Data processing section). These intensities were set at 110%, 125%, 145%, or 160% of the participants’ VT. On day 3, participants completed one additional LMCL bout and two additional HSCL bouts. Participants were required to lie supine for 15 min between the two HSCL bouts. On day 4, participants completed one additional LMCL and one HSCL bout.

The LMCL tests consisted of a period of 2 min of rest upon the cycle ergometer where gas exchange data were collected, followed by 2 min of unloaded cycling and 6 min of cycling at a constant power output. Following each LMCL test, participants were required to lie supine for a period of 10 min to mitigate any risk of adverse events. The HSCL tests also required participants to rest for 2 min on the cycle ergometer to collect gas exchange data to ascertain the quality of the calibration, followed by cycling at the predetermined exercise intensity. For all HSCL tests, Watts values were programmed into the electronic ergometer prior to the start of each bout, were applied automatically once the cadence exceeded 35 rev·min^-1^, and required ∼3 s to attain the set target value. Participants exercised until exhaustion, verified by the inability to sustain cadence within 10 rev·min^-1^ from their chosen target. For safety reasons, prior to allowing the participants to leave, participants were also required to lie supine for 15 min after their final HSCL test.

Throughout all trials, participants were kept unaware of the exercise intensity and elapsed time but were provided visual awareness of their cadence. When participants returned on day 3, they completed two HSCL trials and 1 LMCL trial (65% of W_peak_), and on the fourth testing day, subjects completed one remaining HSCL test and one LMCL test (75% of W_peak_).

### Measurements

#### Pulmonary gas exchange measurements

All exercise tests were completed using an electronically braked cycle ergometer (Excalibur Sport, Corval Lode B.V., Lode Medical Technology, Groningen, the Netherlands), with gas exchange data being collected using a compliant and elastic mixing bag connected to the expired side of the mouthpiece. Expired air was constantly pumped from the mixing bag to rapid-response electronic gas analyzers (AEI Technologies, Model S-3 A and Model CD-3H, Pittsburg, PA, USA) and sampled from the analyzers for 100 m at the start of each new inhalation using a data acquisition system (National Instruments, Austin, TX) controlled by custom software (LabVIEW™, National Instruments, Austin, TX). Ventilation was measured from integrated air flow using a fast-response turbine flow transducer (Hans Rudolph-430, Van Nuys, CA, USA) connected to the inspired side of the mouthpiece and integrated into the same data acquisition system and custom software. The flow turbine processed air flow signals so that tidal volumes were included as an output analog signal (Kim and Robergs, 2012). The calibration of the breath-by-breath system was conducted prior to the ramp test using a commercial medical-grade calibration gas (room air and then 17.2% O_2_ and 4.13% CO_2_) and a 3-L syringe.

### Data processing

The detection of the subjects’ VT was necessary to determine the appropriate HSCL exercise testing intensity. The detection of the subjects’ VT was established using the ventilatory equivalents method with custom-made software (LabVIEW™, National Instruments, Austin, TX, USA). This occurred by applying linear segments to three areas and subsequently adjusting them to the lowest residual error. VT was determined as the time of intersection between the baseline response (slope ∼0), also known as segment 1, and the initial deviation from baseline, also known as segment 2. The detection of this intersection required the agreement between two investigators (±10 s). This measure and method were detailed, compared to other methods, and validated by Caiozzo et al. (1982). In more recent years, the VT has been referred to as the gas exchange threshold (GET) that occurs prior to the second ventilatory breakpoint (the respiratory compensation point, RCP) (Jamnick et al., 2020; Poole et al., 2021).

The subjects’ peak power (W_peak_) was useful for the determination of the correct intensity for the LMCL exercise test (Equation 5).

$${Watts}_{peak}=\left(tte-resting\;time\right)\times ramp\;function,$$ (5)

where tte = time to exhaustion; resting time = total time taken for resting gas exchange data to be met; ramp function = the pre-determined ramp Wattage increment (Watts·min^-1^) from the test of $\dot{V}$ O_2max_.

All data cleaning and processing was carried out using custom-developed software (LabVIEW™, National Instruments, Austin, TX, USA), in which breath-by-breath variability in the data (caused by variation in tidal volume and breathing frequency) were restrained by applying a 7-breath moving average for all data. $\dot{V}$ O_2max_ was defined as the highest 7-breath averaged data point from the incremental ramp exercise protocol.

CP was quantified from the data for TTE and Watts from the four HSCL exercise bouts of each participant. The calculation of CP was first based on graphing the TTE (y-axis) to exercise intensity (Watts) (x-axis) using commercial graphics and curve fitting software (GraphPad Prism, V10, Boston, MA, USA). The graphical data were then fit with a one-phase exponential decay (Equation 1). The CP was computed as the Watts at the decay plateau response resulting from the increasing TTE across lower exercise intensities (Watts). For the calculation of the curvature constant (W′), the data were transformed to reciprocal values, followed by application of linear regression, where the slope of the linear response equated to W’ (Equation 6) (Jones et al., 2010; Poole et al., 2016).

$$Y=Span\;.\;e^{-k.x}+Plateau.$$ (6)

Curve fitting for all relevant phases of the full VO_2_ dataset of each bout was also conducted using added features of the custom-developed software, in which all traditional phases were named and identified [Phase 1, Phase 2, Linear Onset (LO), Total Mono-exponential, Remainder Mono-exponential (the data from the end of the LO segment to the end of the data), and (if applicable) Slow Component]. For the purposes of this manuscript, only the LO-phase data were analyzed for all exercise bouts. For steady-state exercise bouts, mono-exponential analyses were also performed. Steady-state $\dot{V}$ O_2_ was verified by the absence of an increased $\dot{V}$ O_2_ response during the last 4 min of data (linear regression slope not statistically different from zero).

The LO segment data slope was identified by the removal of the Phase 1 data, which, by definition, indicated the beginning of the Phase 2 segment. The Phase 1 data were detected through an exaggerated kinetic response that leveled to the then sustained increase in $\dot{V}$ O_2_. If this response was present, it was deleted, and this typically was constrained to the initial 15–25 s of the data. If Phase 1 was not detectable, no data adjustment was carried out. Now that the beginning of the Phase 2 response was identified, the following data segment was increased one data point at a time, with added adjustment to the first data point, until the segment and accompanied linear regression fit had the lowest residual error. For exercise bouts where a mono-exponential function was applied, the Phase-2 data segment was fitted with the traditional mono-exponential function (Equation 4). The residual error of the linear vs. mono-exponential fit for the LO segment was then quantified based on the SE using previously mentioned commercial curve-fitting software.

**FIGURE 3:**
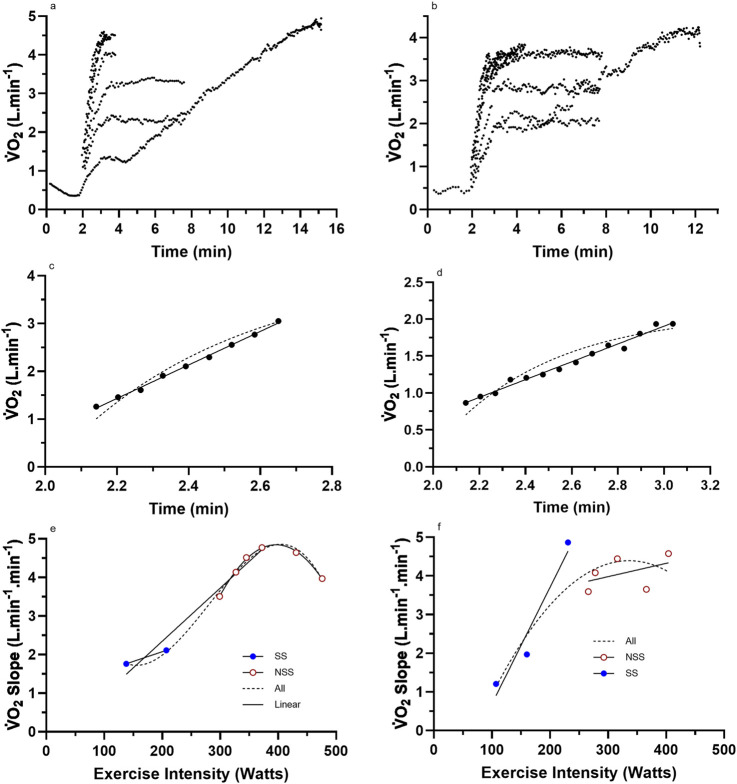
Representative data from two subjects for **(a,b)** the nine (incremental exercise to $\dot{V}$ O_2max_ + eight constant intensities) cycling bouts; **(c,d)** an example bout to show the difference in linear vs. mono-exponential fit for the LO segment; **(e,f)** the total processed LO slope data for each subject across the eight different constant-intensity exercise bouts. For these two subjects, there was an inability to sustain a linear increase in the $\dot{V}$ O_2_ slope of the LO segment with increases in the exercise intensity into the severe domain. NSS, non-steady-state exercise bouts; SS, steady-state exercise bouts; All, linear or non-linear regression of the combined dataset.

Graphs were fitted for all subjects for the LO slope (y-axis) vs. the exercise intensity (%CP) (x-axis) from each of the LMCL and HSCL exercise bouts. Upon the completion of the LO slope analysis, each subject’s results were analyzed to gauge the general linearity vs. non-linearity of their results. Based on an increasing vs. stable or decreasing slope across the last three data points, subjects were then placed into either a “continuous” or “plateau” category (see Figures 2, 3), whereby subsequent analyses could assess the significance of group differences for pertinent variables (See *Results*).

### Statistical analyses

Mean data statistical analyses were completed using IBM SPSS Statistics for Windows (Version 26.0. Armonk, NY: IBM Corp). Such analyses included a Shapiro–Wilk test for documenting the normality of the data for the dependent variables of exercise intensities expressed as %CP, the LO segment SE, for both linear and mono-exponential fits for the initial five exercise bouts (four LMCL and initial HSCL), and the linear slopes of the LO segment for all LMCL and HSCL bouts for the subjects of both the “continuous” or “plateau” groups.

A one-way repeated-measures ANOVA was used to document the extent of differences between the eight different exercise intensities. A two-way repeated-measures ANOVA was used for the SE of the LO segment across exercise intensity (five levels: four LMCL and initial HSCL bouts expressed as %CP) vs. curve fitting (two levels: linear vs. mono-exponential). A mixed-design (between–within) two-way ANOVA was used for the LO slopes across all exercise intensities (eight levels: four LMCL and four HSCL bouts expressed as %CP) vs. GROUP (two levels: “continuous” vs. “plateau”). Significance for both the one- and two-way ANOVAs was observed through the generic sphericity-assumed trait. For both two-way ANOVA analyses, if there was a non-significant interaction, main effects were interpreted. For the mixed-design two-way ANOVA, a significant interaction effect was followed by *post hoc* analyses using the Tukey test. The difference between “continuous” vs. “plateau” groups for selected variables of cardio-respiratory and muscular endurance fitness was investigated using one-sided unpaired t-tests.

## Results

### Descriptive characteristics

The descriptive data for the subjects are presented in Tables 1, 2. Note that despite the relatively high level of training of the participants, there was a large range in $\dot{V}$ O_2max_ (Table 1) and both absolute and relative expressions of the VT and CP (Table 2). The ramp protocols for measurement of $\dot{V}$ O_2max_ varied between 30 and 40 W⋅min^-1^ across the subjects, and time to exhaustion for the $\dot{V}$ O_2max_ ramp protocols was 11.38 ± 1.65 min (7.42–14.13 min).

**TABLE 2:** Added variables from the incremental exercise test.

Subject #	Test (min)	VT (Watts)	VT (%pWatts)	CP (Watts)	CP (%pWatts)	CP (%VTWatts)
1	13.14	340	73.91	374	81.30	110.00
2	11.28	357	79.16	389	86.25	108.96
3	11.42	230	57.50	231	57.75	100.43
4	14.13	289	58.38	317	64.04	109.69
5	10.12	230	64.97	254	71.75	110.43
6	12.10	304	62.81	326	67.36	107.24
7	7.42	134	60.09	146	65.47	108.96
8	12.35	261	60.42	273	63.19	104.60
9	13.16	298	64.64	322	69.85	108.05
10	10.15	253	71.27	276	77.75	109.09
11	10.63	332	78.12	352	82.82	106.02
12	11.79	337	71.40	358	75.85	106.23
13	11.10	260	67.01	229	59.02	88.08
14	10.57	254	68.65	260	70.27	102.36
Mean ± SD	**11.38** ± **1.65**	**277** ± **59**	**67.02** ± **7.03**	**293** ± **67**	**70.91** ± **8.87**	**105.73** ± **5.87**

pWatts = Watts at $\dot{V}$ O_2_ *max*.

### Representative subject data

Raw and processed $\dot{V}$ O_2_ slope data for four representative participants are presented in Figures 2, 3. The data reveal the $\dot{V}$ O_2_ responses of the subjects for each of the eight exercise bouts (Figures 2a,b, 3a,b), examples of the linear vs. mono-exponential curve fitting for the LO segment (Figures 2c,d, 3c,d), and the profiles of the LO slopes across the eight different exercise intensities (Figures 2e,f, 3e,f). Note the increasing LO segment slopes (kinetics) for each bout of increasing exercise intensity. The presence of linear segments for the initial 2-min stage of the incremental protocol is also noted, which is somewhat complicated by the need to remove the initial exaggerated kinetics of Phase 1 and how these responses differ between the subjects. The differences in the LO segment for linear vs. mono-exponential functions are obvious for the data processing examples in Figures 2c,d, 3c,d. Finally, the different profiles of the LO $\dot{V}$ O_2_ segment slopes for the subjects in Figures 2e,f, 3e,f document an unexpected finding from this research, revealing two subset groups of the participants based on the profiles of the LO slopes across the eight exercise intensities. The evidence and explanation for the categorization of the subjects into two groups will be progressively provided in the content to follow.

It is important to document the linearity of the initial segment. We refer to this as the linear onset (LO) segment, but traditional terminology would label it as the initial segment of the Phase 2 response. This linear segment across all subjects and exercise bouts (=112 data sets) consisted of 6–24 data points (=breaths) with a mean ± SD = 10.2 ± 3.0. Of the 112 datasets, 111 had statistical significance (slope different to 0.0) of p < 0.0001, while the remaining dataset had significance at p = 0.0003. This segment is clearly linear, is highly consistent across all subjects and exercise intensity trials, and consequently, represents a different physiological entity to the mono-exponential dependence of the traditional Phase 2 dataset (see the next sub-section and *Discussion*). From a time perspective, the data for the LO segment for mean ± SD and range (min–max) of the start (exercise commenced at 2 min) and end times, and then segment duration and range, were 2.1 ± 0.16 min (1.75–3.18 min), 2.54 ± 0.18 min (2.16–3.44 min), and 0.44 ± 0.15 (0.22–1.08 min), respectively.

The exercise intensities of the eight different bouts were expressed relative to each subject’s critical power, and these values were analyzed using repeated-measures one-way ANOVA. Results revealed an overall significance (F = 885.451; df = 1, p < 0.001), with each level of INTENSITY being significantly different (p < 0.001). Mean ± SD results for these data are 42.94 ± 5.35, 64.37 ± 8.04, 92.97 ± 11.55, 98.79 ± 10.99, 110.47 ± 9.72, 115.96 ± 11.24, 134.41 ± 12.99, and 148.36 ± 14.30 %CP. Although this analysis was expected to reveal the significance between each exercise condition, the core purpose was to quantify the percentage of the CP for each exercise condition so that the responses of the LO $\dot{V}$ O_2_ slope datasets could be better interpreted.

### Linear onset vs. mono-exponential method

**FIGURE 4:**
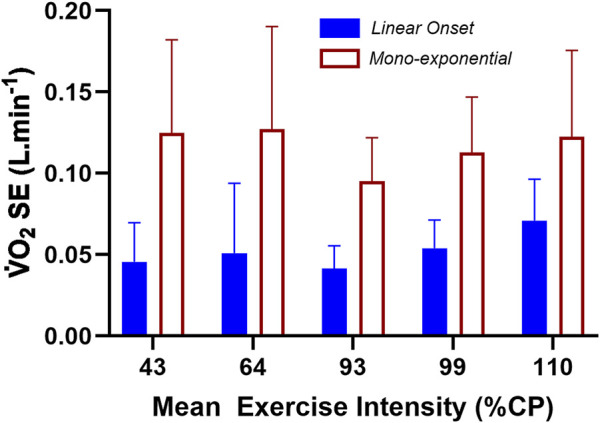
The standard error of the estimate (SE) results for the linear fit of the LO segment vs. the forced mono-exponential fit of the total $\dot{V}$ O_2_ dataset applied to the LO segment. See *Methods* for details. Method differences for all exercise intensities were significant (see *Results* text explanations).

The main research question concerned the comparison of the linear regression results of the LO segment to the traditional mono-exponential fit to the entire dataset of the steady-state bouts applied to the LO segment. This could not be done for the highest three exercise intensities as the $\dot{V}$ O_2_ response was not suited to mono-exponential profiling (see Figures 2a,b, 3a,b). As such, the data of the SE of the LO segment for both methods, presented for Figure 4 have five levels of intensity. All pertinent $\dot{V}$ O_2_ datasets (n = 10 for the SE ANOVA) were first tested for normality using the Shapiro–Wilk test. As only two of the 10 datasets deviated from normality, to ensure protection against a type-II error, the total data were processed statistically as meeting the requirement of normality.

Results from the repeated-measures two-way ANOVA of the SE data revealed a non-significant INTENSITY main effect (F = 1.979; df = 4; p = 0.111), a significant METHOD main effect (F = 99.273; df = 1; p < 0.001), and a non-significant INTENSITY × METHOD interaction (F = 2.053; df = 4; p = 0.100). Based on the SE of the linear vs. mono-exponential fit of the LO segment data, there was a significantly lower residual error when applying linear-onset kinetics, regardless of the exercise intensity or whether the constant-load bouts were steady-state or non-steady-state.

### Group data based on linear vs. non-linear $\dot{V}$ O_2_ onset slope responses

For the subjects of all Figure 2 subset data, the data responses revealed consistent linear responses for the LO $\dot{V}$ O_2_ slope data across all exercise intensities (steady state and non-steady state) (see Figures 2e,f, 3e,f). This differed to the subjects’ data for all (Figure 3) subsets, which revealed a clear non-linearity in the LO $\dot{V}$ O_2_ slope data across the higher exercise intensity conditions. These trends were further explored across all subjects based on the individual subject data for the profile of the $\dot{V}$ O_2_ LO slopes (y-axis) expressed across the exercise intensity (Watts; x-axis). Based on this grouping process, subjects were able to be categorized as either linear (n = 7) or non-linear responders (n = 7). Such subject grouping is presented in Table 3.

**TABLE 3:** Subject characteristics for continuous vs. plateau grouping.

Subject	$\dot{V}$ O_2Max_ (L.min^-1^)	$\dot{V}$ O_2Max_^∼^	$\dot{V}$ O_2Max_ (Watts)	VT (Watts)	CP (Watts)	Linear slope*	Peak linear intensity
*Continuous*							
1	5.9	67.41	460	340	374	0.01186	543
2	5.69	76.84	451	357	389	0.01592	571
4	5.41	63.38	495	289	317	0.01233	462
6	5.91	60.74	484	304	326	0.00451	486
7	4.97	43.38	432	261	273	0.01146	214
11	4.42	57.57	425	332	352	0.01221	531
14	4.85	52.88	370	254	260	0.00972	407
Mean ± SD	4.92 ± 1.30	60.31 ± 10.67	415 ± 94	287 ± 76	309 ± 83	0.0114 ± 0.0035	459 ± 121^#^
*Plateau*							
3	5.09	73.14	400	230	231	0.009878	287
5	3.8	54.71	354	230	254	0.007977	266
8	2.24	60.55	223	134	146	0.01312	281
9	4.94	55.6	461	298	322	0.01378	372
10	4.24	59.56	355	253	276	0.03013	278
12	4.87	71.54	472	337	358	0.01719	422
13	4.33	51.70	388	260	229	0.003574	286
Mean ± SD	4.61 ± 0.48	60.97 ± 8.330	409 ± 48	267 ± 38	278 ± 48	0.0134 ± 0.0085	313 ± 59

^See Table 1 for subject numbers; ∼ mL.kg^-1^.min^-1^; *L.min^-1^.min^-1^.Watt^-1^; #p < 0.05 vs. Plateau group.

An additional mixed-design (between–within) ANOVA was run for the LO $\dot{V}$ O_2_ slope datasets. Such an analysis compared Group (Continuous vs. Plateau responders; two levels; n = 7 vs. n = 7, respectively) and INTENSITY (mean exercise intensity, %CP, eight levels). The results revealed a significant GROUP main effect (F = 6.039; df = 1, 48; p = 0.0177), a significant INTENSITY main effect (F = 13.19; df = 7, 48; p = <0.0001), and a significant GROUP x INTENSITY quadratic interaction (F = 3.75; df = 7, 48; p = 0.033) but a non-significant linear interaction (F = 2.281; df = 7, 48; p = 0.157). The data are presented in Figure 4.

These systematic (non-random) responses reveal the presence of complex physiology that has yet to be identified in prior research on $\dot{V}$ O_2_ kinetics based on the traditional mono-exponential method. The traditional method is confined to exercise increments to steady state and being based on a mono-exponential function assumes that there are no different phases, or components, of this response. These results are elaborated below and further interpreted in *Discussion*.

**FIGURE 5:**
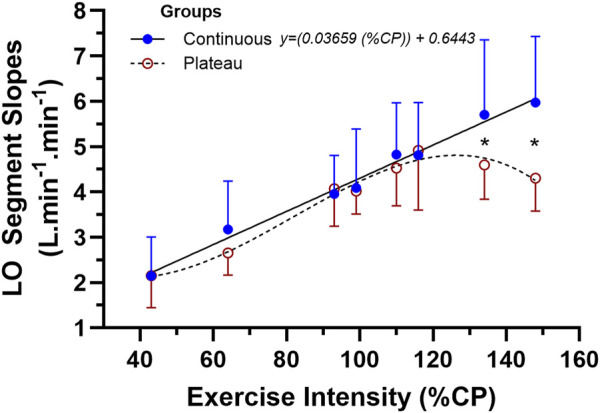
The mean ± SD data for the two-way mixed-design ANOVA for the linear-onset $\dot{V}$ O_2_ slope data between subjects of the continuous vs. plateau group responders across the eight levels of exercise intensity. * represents the location of the interaction effect for the final two levels of exercise intensity (p < 0.05).

The ANOVA results and subsequent Figure 5 show the similarity in LO $\dot{V}$ O_2_ kinetics and low- to moderate-intensity exercise bouts between subjects of the two groups, but the statistically significant deviation in kinetics for the highest two exercise intensities for subjects of the plateau group. As such, it is proposed that the quadratic interaction effect from the ANOVA is valid and that the multiple-comparison-corrected Tukey test for simple comparisons between groups at exercise intensities of 134 (p = 0.0297) and 148 %CP (p = 0.0015) reveals a clear discrimination in the LO $\dot{V}$ O_2_ kinetics between the two groups at such highest exercise intensities.

Some of the additional descriptive data collected during this study could assist in explaining the marked group differences in LO $\dot{V}$ O_2_ kinetics at this higher exercise intensity. The results for the two-sided unpaired t-tests between the two groups for each of the variables (t value, df, and p-value) were as follows: $\dot{V}$ O_2max_ (L.min^-1^) (0.5921, 12, and 0.565), $\dot{V}$ O_2max_ (mL.kg^-1^.min^-1^) (0.1204, 12, and 0.960), $\dot{V}$ O_2max_ Watts (0.1644, 12, and 0.872), VT watts (0.6266, 12, and 0.543), CP watts (0.8697, 12, and 0.402), linear slope (0.586, 12, and 0.569), and peak linear intensity (2.86, 12, and 0.014). None of the variables that quantify musculoskeletal and cardiorespiratory endurance differed between groups, with only the peak intensity of the linear segment of the LO $\dot{V}$ O_2_ slope profile being significantly larger for the continuous profile group vs. the plateau group. There is something uniquely different between the subjects of both groups, independent of endurance fitness, which supports a sustained high LO $\dot{V}$ O_2_ kinetics into severe exercise intensities.

These are a surprising collection of results given the relatively homogenous subject sample, the unique nature of the data processing methods of this research, and the ability of the linear-onset $\dot{V}$ O_2_ kinetics method to clearly discriminate two very different sub-groups within the relatively homogenously trained subject sample. The findings are elaborated in *Discussion*.

## Discussion

### Overview

This study involved two components. The first component compared the linear vs. mono-exponential fit of the LO data segment (∼1 min) $\dot{V}$ O_2_ datasets to establish the model that best fits the data. For all subjects, the linear fit of the LO $\dot{V}$ O_2_ data segment had significantly less error (SE) (between two- and three-fold lower) for the linear vs. mono-exponential model. The results are similar to the prior results of McNulty and Robergs (2017) and document the inadequacies of the traditional mono-exponential model for this segment of the $\dot{V}$ O_2_ response to an exercise transition.

An added purpose was to further explore the utility of the LO $\dot{V}$ O_2_ kinetics method to document changing $\dot{V}$ O_2_ kinetics with increases in the exercise intensity, regardless of whether the exercise conditions were steady or non-steady state. Interestingly, upon analyzing individual differences across subjects for the profile of the change in the linear slope of the LO $\dot{V}$ O_2_ data for different bouts of exercise, it was observed that not all subjects exhibited a consistent linear increase with increasing exercise intensity. All subjects exhibited similar increases in the linear slope of the LO $\dot{V}$ O_2_ data segment across the initial six exercise intensities spanning both steady- and non-steady-state exercises (43–116 %CP). However, half of the subjects showed a decline in the LO $\dot{V}$ O_2_ slope at the two highest intensities (134 and 148 %CP), suggesting that physiological or even possibly pathophysiological factors can account for individual differences. Of additional interest was the finding that the two groups of subjects (continuous slope increase vs. plateau slope response) were not different on multiple measures of cardiorespiratory endurance fitness. The results are further explained in the *Discussion*.

### $\dot{V}$ O_2_ kinetics of exercise transitions to a higher steady state

As previously introduced, the traditional mono-exponential model of the $\dot{V}$ O_2_ kinetics during exercise transitions to a higher steady state depends on the total $\dot{V}$ O_2_ dataset of exercise bouts lasting anywhere from 5 to 10 min. The methodology constrains the study of $\dot{V}$ O_2_ kinetics to steady-state exercise intensities and, as such, does not view any subset data segment to have independent physiological meaning or importance. This is unfortunate because it has precluded the initial $\dot{V}$ O_2_ response to an exercise condition from kinetic analyses when there is clearly a kinetic response based on the rate of change in $\dot{V}$ O_2_ (dx/dt; where x = $\dot{V}$ O_2_). This kinetic response occurs for all exercise intensities (steady and non-steady state), is not dissimilar to the research on onset kinetics responses such as enzyme kinetics and muscle contractile force production, and therefore warrants research inquiry.

The added importance of an additional feature of LO $\dot{V}$ O_2_ kinetics research (the initial component of the Phase 2 response based on the traditional terminology) is that it removes the complication of the presence of a $\dot{V}$ O_2_ slow component or the more abrupt cessation of exercise after 1 minute of exercise due to contractile failure. We will focus on the unique data and related physiology presented within the *Results* section.

### The reduced residual error of linear-onset $\dot{V}$ O_2_ kinetics

McNulty and Robergs (2017) reported a statistically significant reduced SE for the linear regression model of the LO $\dot{V}$ O_2_ data (∼first min) of the exercise bouts. This pattern also extended to a non-linear fit of the $\dot{V}$ O_2_ remainder segment (end of the LO segment to the end of the dataset) with a significantly reduced SE compared to the total dataset mono-exponential fit for intensities beyond 45% of the subjects’ VT. Such prior results are now further reinforced by the findings of this study.

Across the five lowest exercise intensities (43, 64, 93, 99, and 110 %CP), the $\dot{V}$ O_2_ responses were suited to mono-exponential profiling. However, for the higher exercise intensity conditions (116, 134, and 148 %CP), there were more abrupt increases in $\dot{V}$ O_2_ with no evidence of a mono-exponential function transitioning into a steady state or $\dot{V}$ O_2_ slow component. Consequently, for the five lowest exercise intensity bouts where both LO and mono-exponential curve fitting (total dataset or to the start of a $\dot{V}$ O_2_ slow component) could occur, there was a significantly lower SE for the linear regression model as opposed to the traditional mono-exponential (non-linear) fit. Hence, regardless of the exercise intensity, or whether each trial was steady-state or not, there was a significantly reduced SE when the LO $\dot{V}$ O_2_ kinetics method was applied. These results now expand upon the earlier study (McNulty and Robergs, 2017) to incorporate non-steady-state conditions. Additionally, in all the respective exercise intensity conditions, the LO $\dot{V}$ O_2_ kinetic response can be viewed in a systematic and non-chaotic form, which indicates that the causes of these responses are not erroneous and therefore involve complex physiology that requires further research and understanding.

### Explanation of the grouped differences in linear-onset $\dot{V}$ O_2_ kinetics

The results provided in Table 3 and Figures 2, 3, 5 indicate that some subjects can maintain a continual linear increase in their LO $\dot{V}$ O_2_ kinetic response across a wide range of exercise intensities, and others cannot. These unique results stimulate curiosity surrounding the physiological differences that lead to these contrasting responses. Subject recruitment processes were designed with the intent of creating a similar profile among subjects regarding training habits and related endurance fitness levels. Thus, even though there were differences in measures of cardiorespiratory and muscular endurance between the sample subjects (see Tables 1, 2), both groups contained subjects with high and lower $\dot{V}$ O_2max_, VT, and CP data. For example, from Table 2, it is noted that subjects 1, 2, 4, 7, and 14 belong to the continuous group and subjects 3, 12, and 13 to the plateau group. Clearly, for this subject sample, the LO $\dot{V}$ O_2_ kinetics method shows a remarkable ability to discriminate clear differences within a sample of moderate to highly endurance trained cyclists, with the added ability to discriminate between athletes within the sample that were all of elite endurance capacities (subjects 1, 2, 3, and 12).

**FIGURE 6:**
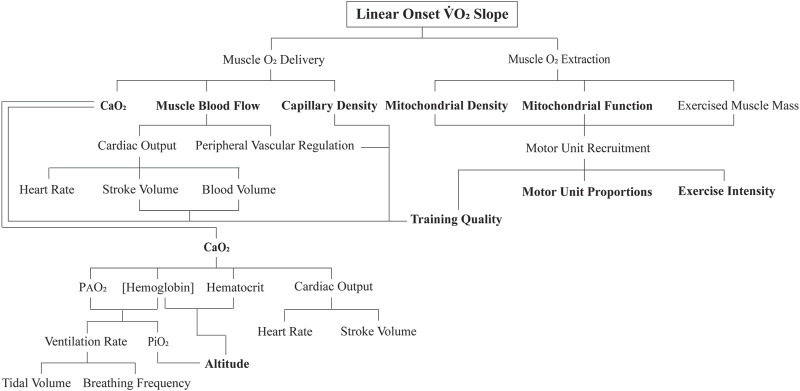
Flow diagram of the proposed key physiological determinants to the linear onset (LO) $\dot{V}$ O_2_ kinetics segment. The sub-components in bold are proposed to be the main independent variables of interest to future research inquiry.

What aspects of exercise physiology could account for these results? This exploratory study discovered highly original findings and therefore was not designed to answer this question. Future research studies need to document these physiological determinants; for now, we can only use knowledge and rationale thinking to speculate potential mechanisms. To commence this process, it is best to formulate a flow diagram of the physiology that could influence the LO $\dot{V}$ O_2_ segment (Figure 6).

To begin with, applying a higher exercise intensity for an exercise transition necessitates an increase in motor unit recruitment. During such an increasing skeletal muscle metabolic demand, there is a subconscious refinement to recruit more motor units (Hug et al., 2022). As best as we can tell from animal research, humans recruit motor units in a similar-size principle-governed manner, with slow-twitch categories of motor units recruited first, followed by a progressive additive recruitment of intermediate-twitch motor unit categories, and eventually the recruitment of pure fast-twitch glycolytic motor units (Mendell, 2005). This increasing active muscle mass, accompanied by an increasing rate of contraction frequency or contraction force for a given frequency (pertinent for this study as each subject was required to maintain the same cadence for all exercise bouts) does not require large changes in cellular metabolic regulation (Petajan, 1991). The added contracting muscle fibers add more units of $\dot{V}$ O_2_, so with each bout of increased exercise intensity, the rapid recruitment of added motor units would account for a faster LO $\dot{V}$ O_2_ kinetic response (Jones et al., 2004).

As observed in Figure 5, for the low- to moderate- and then initially non-steady-state exercise intensities, the subjects of both groups of responders had similar multiple bout LO $\dot{V}$ O_2_ kinetic profiles. As such, it is reasonable to assume that the LO $\dot{V}$ O_2_ kinetic response is highly motor unit recruitment-driven. It would be interesting to see whether, as documented in Figure 6, alterations in the inspired partial pressure of oxygen (via increased altitude or inhalation of hypoxic gas) changes these responses or whether clinical populations that differ in myocardial function, hematology, lung function, skeletal muscle mass, etc., would also have constrained LO $\dot{V}$ O_2_ kinetics for increasing exercise intensity transitions. It is, therefore, logical to conclude that the deviant LO $\dot{V}$ O_2_ kinetic responses of the plateau group subjects could be linked to increasing recruitment of fast-twitch motor units, which based on training protocols could be less aerobically adapted (see below), and as such, impart slower LO $\dot{V}$ O_2_ kinetics for the heavy- and severe-domain exercise intensities. In addition, and as proposed by Adami et al. (2011) and Keir et al. (2016), there could be constrained increases to the regional distribution of intramuscular capillary blood flow as fast-twitch motor units are recruited, which is further exaggerated by the associated increased heterogeneity in each of muscle metabolic demand, oxygen delivery, and cellular $\dot{V}$ O_2_.

A potential explanation within the context of motor units could be the influence of the different genetics of the subjects to the trainability of their muscle metabolic, cardiovascular, and neuromuscular systems. Numerous studies have investigated the genomic differences between individuals that may account for changes in $\dot{V}$ O_2_ kinetics. Bouchard et al. (2011) conducted a genome-wide association study investigating over 300,000 single-nucleotide polymorphisms (SNPs) and found that carriers of a specific SNP (rs6552828) can account for up to ∼6% of the variance in the $\dot{V}$ O_2max_ response. Furthermore, stepwise multiple regression analysis of the 39 SNPs identified a subset of 21 SNPs that explained 49% of the variance in $\dot{V}$ O_2max_ trainability. Subjects possessing nine or fewer favorable alleles at these 21 SNPs exhibited an increase in $\dot{V}$ O_2max_ of 221 mL⋅min^-1^, whereas those with 19 or more favorable alleles demonstrated an average improvement of 604 mL⋅min^-1^ (Bouchard et al., 2011). Although these results show promise for the role of genetics in how we understand $\dot{V}$ O_2max_ data, its application to other exercise intensities, such as bouts of constant-intensity exercise, may be questionable and, as such, should be interpreted with caution.

Although the intent of subject recruitment was to create a comprehensive profile on highly trained subjects, individual physiological adaptations that occur as a result of training intensity may still differ between subjects, which, in turn, can account for significant differences in $\dot{V}$ O_2_ responses between subjects. One such change that has recently gained attention is that of mitochondrial adaptation. Although there is an undisputed importance of mitochondrial content and function in exercise physiology, whereby the respiratory capacity of the mass-specific skeletal muscle was identified as the optimal determinant of endurance performance, there is some debate as to the ideal training conditions to optimize both (Bouchard et al., 2011). Bishop et al. (2014) investigated the optimal training prescription to achieve both variables and found that training at an intensity above a moderate level (>65% HRmax) was the most beneficial in observing significant improvements in mitochondrial function (as observed through mitochondrial respiration efficiency). In contrast, training volume had the most beneficial effect in improving mitochondrial content (as observed through citrate synthase activity).

These results were further reinforced by that of Lundby and Jacobs (2016) where they confirmed that exercise-induced adaptations conducive to improved mitochondrial function are dependent on the intensity of the training and are most likely explained by the improved expression of mitochondrial enzymes that accelerate aerobic metabolism. In this study, subjects were recruited based on a loose training volume measurement (minimum of 3 × 45 min cycling sessions per week), whereby the training intensity was overlooked. Therefore, while meeting our predetermined standard of “highly trained,” the underlying physiological adaptations as a direct result of the exercise they had undergone, specifically, their skeletal muscle mitochondria function, might not have been entirely indicative of a high endurance performance. Hence, the contrasting responses of the LO $\dot{V}$ O_2_ slopes of Figure 5 may be potentially explained by the poorly adapted mitochondrial function of the muscle fibers of the intermediate fast-twitch motor units, and thus, the lower $\dot{V}$ O_2_ kinetics, independent of capacity (e.g., $\dot{V}$ O_2_max, VT, and CP), of those subjects.

Despite the prior research evidence, the results of this study are clear in revealing that the physiological determinants of the between-subject variability in the LO $\dot{V}$ O_2_ kinetics for high- to severe-intensity exercise bouts are not associated with components of $\dot{V}$ O_2max_ trainability. However, the previously found content on the function and adaptive responses of muscle mitochondria to different forms of exercise training could be meaningful when accompanied by an understanding of changing motor unit recruitment with increases in the exercise intensity. This added content is also revealed in Figure 6 in the components proposed to be influenced by neuromuscular function and types of exercise training. Individuals who train based on volume and relatively lower-intensity exercise would attain mitochondrial adaptations constrained to the muscle fibers across the slow- to intermediate-twitch motor units. Conversely, individuals who do more quality interval-type exercise training within their mode-specific tasks would stimulate increased mitochondrial density and improved function in all motor units recruited, including the fast-twitch motor units of the intermediate categories that have moderate mitochondrial content (Lundby and Jacobs, 2016; Bottinelli et al., 1996; Bottinelli and Reggiani, 2000).

Such observations and interpretations are particularly pertinent for the method and data we present for LO $\dot{V}$ O_2_ kinetics spanning low- to severe-intensity exercise. Perhaps the limited recruitment of intermediate- and fast-twitch motor units inherent with long slow-distance exercise training constrains muscle metabolic adaptations to the slow-twitch motor unit pool. Progression to non-steady-state exercise then exposes the individual to less endurance trained muscle fibers. A further extrapolation of this interpretation relates to those individuals with more fast-twitch than slow-twitch motor units in their prime muscles for movement patterns of their specific exercise mode. Although these individuals would be naturally directed to more intense or powerful, and hence shorter-duration, exercise and sports, there would be a range of motor unit proportions revealing more even proportions of slow- to fast-twitch motor units in successful moderate-length road cyclists and triathletes. The training effect, as previously described, would also be pertinent to these individuals.

There is growing interest in the modeling of exercise transitions from steady state to non-steady state (Korzeniewski and Zoladz, 2015; Dunst et al., 2025). Although this work is interesting from the perspectives of mathematics, physiology, and real-world applications, it remains constrained in relevance to the LO $\dot{V}$ O_2_ kinetics because it does not address the initial 1.5 min of exercise. Such data, based on the duration from the initiation of exercise, are defined as non-steady state and, due to the linearity of the onset segment, precede the mono-exponential extension to steady state or the VO_2_ slow component (see the next section). As such, and as previously explained, the physiology of VO_2_ LO kinetics requires a fresh new approach for applying multiple system physiology and muscle metabolic biochemistry (the features of Figure 6 and perhaps others not addressed by the authors) to this immediate kinetic response.

### $\dot{V}$ O_2_ slow component occurs in a narrow range of non-steady-state exercise intensities

At the commencement of a constant-load exercise bout ending in steady state, $\dot{V}$ O_2_ increases abruptly and then progresses non-linearly to a dynamically stable (variable but on average constant) steady-state value within a 3-min period (Jones et al., 2011). For exercise intensities that exceed the ability to attain a steady state, the time period over which the non-linear phase occurs increases, causing slowed total kinetics that transition into a sustained slow increase in $\dot{V}$ O_2_ (termed the slow component; SC) that may last for several minutes (Jones et al., 2011). The exact causes of the SC have been debated for several years, and currently no consensus exists for the relative contribution of competing causes such as lowered metabolic efficiencies of muscle contraction, increased energetic costs of lactate removal and metabolism, and increased $\dot{V}$ O_2_ of ventilation, although evidence eludes to the main location of the SC to reside within the contracting skeletal muscle (Korzeniewski and Zoladz, 2015; Jones et al., 2011; Colosio et al., 2020; Poole, 1994; Poole et al., 1991; Zoladź and Korzeniewski, 2001; Whipp, 1994). However, it is important to acknowledge that such inferences are based on correlations and not experimental cause–effect research designs.

In conclusion, based on the results of this study, most of the prior research works on the SC have defined it to be across all non-steady-state transitions in the heavy- to severe-intensity domains (Poole et al., 1991). For example, Jones et al. (2011) defined the $\dot{V}$ O_2_ SC to be associated with constant-intensity exercise above the CP, where “*…. no steady state is achievable but, rather, $\dot{V}$ O*_*2*_ *continues to rise with time until the $\dot{V}$ O*_*2*_ *max is reached …*” (p. 1). For this research, the only exercise conditions that induced a $\dot{V}$ O_2_ SC were confined to between 75 and 130 %CP, and at no point during any bout to failure in which an SC was identified did $\dot{V}$ O_2_ exceed 80% of $\dot{V}$ O_2max_. Furthermore, the $\dot{V}$ O_2_ responses of the subjects to the heavy- to severe-intensity exercise bouts (>130 %CP) were so abrupt that some subjects did not exhibit a SC profile due to the sustained large kinetic response of $\dot{V}$ O_2_ and rapid onset of contractile failure (see Figures 2a,b, 3a,b). Clearly, the SC is a response confined to a narrow range of exercise intensities slightly below to slightly above a person’s CP, or as expressed by Colosio et al. (2020), for exercise intensities constrained to the heavy domain.

#### Limitations

This study has a range of limitations. The study involved highly trained participants aged 18–55 years, limiting its relevance to elite-level endurance athletes, untrained individuals, those with chronic conditions or disorders, and people outside this age group. However, as shown in Tables 1, 2, there was a wide range in data for $\dot{V}$ O_2max_, the VT and CP that could also reveal the wider generalizability of the results. Another limitation is the limited fitness screening of participants, ultimately affecting the validity of results to a “highly” trained population. As stated in *Discussion: explanation of grouped differences,* participants were recruited based on a loose training volume measurement, with no consideration of the training intensity. Consequently, it is possible that the training intensity induced physiological improvements in skeletal muscle fibers (mitochondrial function) across more diverse motor unit categories may not have been consistent among individuals.

The issue of gender-based differences is always pertinent to address given the limited research on female subjects. As only two participants were women, despite efforts to recruit more, there is insufficient evidence to generalize the findings to all women or to be able to explore gender differences in this LO $\dot{V}$ O_2_ kinetics response. Nevertheless, it is worth noting that the two female subjects (participants 5 and 7) had different LO $\dot{V}$ O_2_ kinetics responses across all intensity bouts, resulting in each being in different groups (Continuous vs. Plateau), with the female participant with the higher $\dot{V}$ O_2max_ being in the plateau group. It is interesting that such results conformed to the total trend with the participant sample where physical fitness measures did not explain why certain participants were grouped as they were. Based on the prior *Discussion* content, it is possible that genetic features of a person’s physiology, unrelated to gender-related biology, in addition to the quality of exercise training, may be the most influential determinants of LO $\dot{V}$ O_2_ kinetics.

A significant limitation is that the research was focused on cycling, making it potentially inapplicable to running or other forms of exercise. Multiple tests were conducted on designated test days, and it was assumed that the subjects' moderate to high fitness levels, in addition to the rest periods, were sufficient to ensure adequate recovery before subsequent tests and that prior exercise did not affect subsequent exercise sessions. This issue is reduced by the study’s design, which compared one method to another across multiple data sets. To minimize time-of-day errors, tests were conducted at the same time each day for each participant. The validity of the instruments, such as expired gas analysis indirect calorimetry, was ensured by calibrating the system immediately before each test and using a previously validated custom system of breath-by-breath expired gas analysis indirect calorimetry (Kim and Robergs, 2012).

## Conclusions and recommendations

The study of $\dot{V}$ O_2_ kinetics has a long history, fostering the development of complex pulmonary and whole-body physiological discoveries and innovations. Until recently, a pillar of this topic has been the mono-exponential model applied to a total data set of at least 5 min, spanning the increment in $\dot{V}$ O_2_ through the non-linear response to steady state. However, prior research has presented clear evidence that there is an initial linear $\dot{V}$ O_2_ response that was superior to the mono-exponential model and that this response was consistent across all participants studied (Robergs, 2014; McNulty and Robergs, 2017).

The current research is not only consistent with past results (McNulty and Robergs, 2017) but has also expanded them to include non-steady-state or severe exercise—once again indicating a lower SE of a linear vs. mono-exponential fit during the LO segment. When all the eight, constant-intensity LO $\dot{V}$ O_2_ slopes were collectively graphed for each individual, two responses were clearly identified. Half of the subjects exhibited a marked plateau, or even decrease, in their $\dot{V}$ O_2_ slope as the exercise intensities became heavy to severe, whereas the remaining had a continuous linear-increase profile. Explanations for the two disparate group responses remain speculative but could reveal genetic differences between subjects pertaining to motor unit proportions or the extent of adaption to exercise training. There could also be differences between the two groups in the quality of their training. The ability of LO $\dot{V}$ O_2_ kinetics to discriminate between two sub-groups of athletes is unique and rare, further suggesting that there is more physiology to uncover regarding individual LO $\dot{V}$ O_2_ kinetic responses to constant-intensity exercise bouts, regardless of the exercise intensity.

This study reveals potential for further physiological research into the existence of the LO segment and related kinetics from different researchers and laboratories, applied to different exercise modes, and how interventions (e.g., hypoxia, exercise modes, and exercise training) or causal comparative differences between pre-existing groups (e.g., health status, diseases, altitude acclimation, and physical fitness levels) influence LO $\dot{V}$ O_2_ kinetics. There is also a need to further compare results of the kinetics of exercise transitions between the traditional mono-exponential method and the LO method to establish whether the results provide different indices of kinetics for the same subjects. If they do, it is evidence of the two methods, and their kinetics, measuring different features of physiology.

The results of this research, and the opportunities this creates, are exciting as they present novel opportunities to further explore the physiology of exercise during a wide range of increments in the exercise intensity. An additional benefit of this endeavor is that it reveals the discovery that can occur when science does what it should, which is to always follow evidence, and when evidence of anomalies against conventional understanding exists, to then challenge the convention (Kuhn, 1962). Such is the structure of progress in science and the responsibility of all scientists.

## Data Availability

The raw data supporting the conclusions of this article will be made available by the authors, without undue reservation.
